# Evaluation of the Seroprevalence of Infectious Diseases in 2,445 in vitro Fertilization Cycles

**DOI:** 10.1055/s-0041-1725055

**Published:** 2021-04-15

**Authors:** João Guilherme Grassi dos Anjos, Newton Sergio de Carvalho, Karam Abou Saab, Edward Araujo Júnior, Jaime Kulak

**Affiliations:** 1 Universidade Federal do Paraná, Curitiba, PR, Brazil; 2 Escola Paulista de Medicina, Universidade Federal de São Paulo, São Paulo, SP, Brazil; 3 Universidade Municipal de São Caetano do Sul, São Paulo, SP, Brazil

**Keywords:** fertilization in vitro, human immunodeficiency virus, syphilis, hepatitis B, hepatitis C, human T-lymphotropic virus 1, fertilização in vitro, vírus da imunodeficiência humana, sífilis, hepatite B, hepatite C, vírus linfotrópico T humano 1

## Abstract

**Objective** To evaluate the seroprevalence of positive markers for syphilis, human immunodeficiency virus (HIV) I and II, human T cell lymphotropic virus (HTLV) I and II, and hepatitis B and C among women undergoing in vitro fertilization (IVF).

**Methods** We conducted a retrospective analysis among patients who underwent IVF, between January 2013 and February 2016, and who had complete screening records.

**Results** We analyzed 1,008 patients who underwent IVF, amounting to 2,445 cycles. Two patients (0.2%) tested positive for HIV I and II and none for HTLV I and II. Three patients (0.3%) had positive screening for syphilis, and two (0.2%) had positive hepatitis C antibody test (anti-HCV). A positive hepatitis B virus surface antigen (HbsAg) test was observed in 4 patients (0.4%), while 47 (4.7%) patients were positive for IgG antibody to hepatitis B core antigen (anti-HbC IgG), and only 1 (0.1%) was positive for IgM antibody to hepatitis B core antigen (anti-HbC IgM). The anti-HbS test was negative in 659 patients (65.3%). Only 34.7% of the patients had immunity against the Hepatitis B virus. Patients with an anti-HbS negative result were older than those with a hepatitis B test (anti-HbS) positive result (36.3 versus 34.9; *p* < 0.001).

**Conclusion** The present study showed lower infection rates than the Brazilian ones for the diseases studied in patients undergoing IVF. Only a few patients were immunized against hepatitis B.

## Resumo

**Objetivo** Avaliar a soroprevalência de marcadores positivos para sífilis, vírus da imunodeficiência humana (HIV) I e II, vírus linfotrópicos de células T humanas (HTLV) I e II e hepatite B e C em mulheres submetidas a fertilização in vitro (FIV).

**Métodos** Realizamos uma análise retrospectiva entre as pacientes submetidas a FIV, entre janeiro de 2013 e fevereiro de 2016, e que possuíam prontuários completos.

**Resultados** Foram analisadas 1.008 pacientes submetidas a FIV, totalizando 2,445 ciclos. Duas pacientes (0,2%) apresentaram resultado positivo para HIV I e II, e nenhuma para HTLV I e II. Três pacientes (0,3%) apresentaram triagem positiva para sífilis, e duas (0,2%) apresentaram teste de pesquisa de anticorpos anti-HCV (anti-HCV) positivo. Um teste de antígeno de superfície do vírus da hepatite B (HbsAg) positivo foi observado em 4 pacientes (0,4%), enquanto 47 (4,7%) pacientes foram positivas para anticorpos IgG contra o antígeno de superfície da hepatite B (IgG anti-HbC), e apenas 1 (0,1%) foi positiva para anticorpos IgM contra o antígeno central da hepatite B (IgM anti-HbC). O teste de anticorpos contra hepatite B (anti-HbS) foi negativo em 659 pacientes (65,3%). Apenas 34,7% das pacientes tinham imunidade contra o vírus da hepatite B. Pacientes com resultado negativo anti-HbS eram mais velhas do que aquelas com resultado positivo anti-HbS (36,3 *versus* 34,9; *p* < 0,001).

**Conclusão** Este estudo mostrou taxas de infecção inferiores às taxas brasileiras para as doenças estudadas em pacientes submetidas à FIV. Apenas alguns pacientes foram imunizados contra a hepatite B.

## Introduction

The need for assisted reproductive techniques has increased recently, primarily due to an aging population, as well as to the prioritization of studies, and to financial and marital stability. 1 With the advancing of age of women, there is also a greater cumulative exposure to diseases that cause tubeperitoneal involvement, such as endometriosis, thereby increasing the importance of assisted reproductive techniques for treatment. 2 In addition to the increasing need for assisted reproductive techniques, we have been witnessing a high prevalence of certain infectious diseases, most importantly syphilis, in the general population. 3

In assisted reproduction laboratories, there are concerns about the transmission of viral and bacterial diseases among infertile partners and between couples in the facility. This concern is justified, because the burden and risk involved in the majority of these diseases are very high, some leading to impairments in quality of life and, ultimately, mortality. It has become common practice among fertility clinics worldwide to carry out viral and bacterial screening of sexually transmitted diseases (STDs). 4 In Brazil, human immunodeficiency virus (HIV) I and II, syphilis, human T cell lymphotropic virus (HTLV) I and II, and hepatitis B (hepatitis B test [anti-Hbs]; hepatitis B virus surface antigen [HbsAg]; IgM and IgG antibody to hepatitis B core antigen [anti-Hbc IgM and IgG] and hepatitis C (anti-HCV) are routinely tested for.

Although widespread testing has been performed in this population, little is known about the prevalence of these diseases among women undergoing in vitro fertilization (IVF) treatment. There are no studies from Brazil that have investigated all these tests together in this population. The present study was conducted in a specialized center in southern Brazil and, therefore, aimed to determine the prevalence of positivity of tests performed for STDs among women undergoing IVF procedures.

## Methods

In the present study, we performed a retrospective analysis of women undergoing IVF treatment in a specialized center for assisted reproduction in Curitiba, PR, Brazil, between January 2013 and February 2016. The study was approved by the Ethics Committee on Research of Hospital das Clínicas da Universidade Federal do Paraná (UFPR). Due to the retrospective study design, the need for informed consent was waived.

All patients who completed IVF cycles between January 2013 and February 2016 with complete routine screening records were included. All patients underwent routine laboratory testing at the time of IVF indication. If the patients had to repeat the routine screening during the treatment, the first routine results were taken for data acquisition.

Data on age, number of IVF cycles performed, syphilis serologic testing, anti-HIV I and II testing, anti-HTLV testing, HbsAg, anti-Hbs, anti-Hbs IgM and IgG, and anti-HCV testing were extracted from the records of the patients. All procedures related to laboratory testing were performed in standardized and certified laboratories.

Prevalence confidence intervals were calculated using the exact method proposed by Clopper-Pearson. The student's *t* -test for independent samples was used to evaluate the association between age and number of positive serology results for anti-HbSAg. The calculated statistical power was 99%. Normality was evaluated using the Kolmogorov-Smirnov test. Values of *p* < 0.05 indicated statistical significance. Statistical analyses were performed using IBM SPSS Statistics for Windows, version 20.0 (IBM Corp. Armonk, NY, USA) software.

## Results

A total of 1,008 patients who underwent IVF between January 2013 and February 2016 were included in the present study, and all patients underwent routine laboratory testing. The mean age ( ± standard deviation [SD]) was 35.8 ± 4.9 years old. The age distribution of the women is presented in Table 1 . The total number of IVF cycles was 2,445.

**Table 1:** Age distribution of all women

Age (years old)	*n* (%)
≤ 25	17 (1.7)
26 to 30	144 (14.3)
31 to 35	375 (37.2)
36 to 40	290 (28.8)
40	182 (18.1)
Total	1008 (100)

Three patients (0.3%) had positive syphilis screening test results. Two patients (0.2%) had positive HIV screening results. None tested positive for HTLV. Positive anti-HCV serology results were found in 2 patients (0.2%). A positive HbsAg test result was observed in 4 patients (0.4%). The anti-HbC IgM test was positive in 1 patient (0.1%), whereas the anti-HbC IgG test was positive in 47 patients (4.7%). The positive results obtained for the serology tests are shown in Table 2 .

**Table 2:** Serologic prevalence among 1,008 in vitro fertilization women from a fertility clinic between 2013 and 2016

Serologic testing	Positive *n* (%)
Syphilis	3 (0.3)
HIV I and II	2 (0.2)
HTLV I and II	0 (0)
Anti-HCV	2 (0.2)
HbsAg	4 (0.4)
Anti-HbC IgM	1 (0.1)
Anti-HbC IgG	47 (4.7)
Anti-HbS	344 (34.3)

Abbreviations: anti-HbC IgM, IgM antibody to hepatitis B core antigen; anti-HbC IgG, IgG antibody to hepatitis B core antigen; anti-HbS, hepatitis B test; HbsAg, hepatitis B virus surface antigen; HIV, human immunodeficiency virus; HTLV, human T cell lymphotropic virus.

To estimate the possible seroprevalence of these markers in a larger population (all women undergoing IVF in Brazil), the confidence interval (CI) for the seroprevalence of the markers was calculated. The values are shown in Table 3 .

**Table 3:** Confidence interval of the seroprevalences

Serologic testing	Positive *n* (%)	95%CI *
Syphilis	3 (0.30)	0.06–0.87%
HIV I and II	2 (0.20)	0.02–0.71%
Anti-HCV	2 (0.20)	0.02–0.72%
HbsAg	4 (0.40)	0.10–1.01%
Anti-HbC IgM	1 (0.10)	0.002–0.55%
Anti-HbC IgG	47 (4.7)	3.5–6.2%

Abbreviations: anti-HbC IgM, IgM antibody to hepatitis B core antigen; anti-HbC IgG, IgG antibody to hepatitis B core antigen; anti-HCV, hepatitis C antibody test; HbsAg, hepatitis B virus surface antigen; HIV, human immunodeficiency virus.

* Exact method (Clopper-Pearson)

Among the anti-Hbs-positive patients, 302 (31.7%) had anti-HbS positive and anti-HbC IgG negative; these patients are likely to have acquired immunity to hepatitis B through effective vaccination. The remaining 39 were probably immunized due to contact with the virus (positive anti-HbC IgG).

The confidence interval for the anti-HbS seroprevalence was calculated for the whole sample and for patients who showed a positive result for anti-HbS and a negative result (probable vaccination) or a positive result (probable prior disease) for anti-HbC IgG, respectively. The values are shown in Table 4 .

**Table 4:** Confidence interval of the hepatitis B antibody seroprevalence

Serologic testing	Positive	95%CI *
Anti-HbS	344 (34.3%)	31.4–37.3%
Anti-HbS restricted to negative anti-HbC IgG	302 (31.7%)	28.7–34.7%
Anti-HbS restricted to positive anti-HbC IgG	39 (84.8%)	71.1–93.7%

Abbreviations: anti-HbC IgG, IgG antibody to hepatitis B core antigen; anti-HbS, hepatitis B test.

* Exact method (Clopper-Pearson)

We tested the following hypothesis: the mean age of patients who showed an immune response result against hepatitis B differed depending on the type of result. Patients with an anti-HbS negative result were older than those with an anti-HbS positive result (36.3 versus 34.9; *p* < 0.001). The results of descriptive statistics of age according to anti-HbS are shown in Table 5 .

**Table 5:** Hepatitis B antibody positivity versus age

		Age (years old)				
	*n*	Mean	Minimum	Maximum	SD	*p-value* *
Negative	659	36.3	19.8	56.7	4.8	
Positive	344	34.9	21.3	52.6	5.0	< 0.001

Abbreviation: SD, standard deviation.

* t-Student's Test, *p* < 0.05; Power: 99%

## Discussion

So far, no study has evaluated the seroprevalence of HIV I and II, HTLV I and II, syphilis, and hepatitis B and C in patients undergoing IVF. However, one study evaluated the prevalence of syphilis only in couples who underwent assisted reproduction treatment. 5 The importance of the present study is reinforced by its important external validity, reiterated by the number of patients analyzed ( *n* = 1,008), which is a massive sample for a procedure performed in such a restricted population.

Age stratification followed a normality distribution, but with a deviation to the right of the normality curve; this was demonstrated by a mean age of 35.8 ( ±  4.9) years old and a median of 35.6 years old, varying between 19 years and 10 months old and 56 years and 9 months old. This variation is due to the characteristics of the female population studied, along with the increase in the involvement of factors causing infertility over time. 6

The results for the seroprevalences observed in our study were lower than those found in two similar studies, one conducted in Ghana, Africa, and the other in London, England. In the African study, the seroprevalence of HIV, hepatitis B, and hepatitis C was evaluated, with a prevalence of 1.7%, 7.9%, and 0.4%, respectively. The prevalence described in the IVF population was similar to that reported for the general prevalence in Ghana. This study presents the particularity of having been performed in an African country, with seropositivity rates for diseases such as HIV and hepatitis B well above the world averages. 7 In the London study, the seroprevalence of HIV, hepatitis B, and hepatitis C were 0.13%, 1.7%, and 0.5%, respectively. The data were also comparable to that reported for the general population of that country. 8

The seroprevalence of syphilis in patients undergoing assisted reproductive techniques was previously studied by Cavalcante et al. 5 in Goiânia, GO, Brazil, covering patients of high and low complexity in a public hospital and in a private center. In this study, among the 253 female patients who underwent venereal disease research laboratory (VDRL) examination in the public hospital, there was not a single case of syphilis diagnosis. Of the 896 patients surveyed at the private center, only 1 (0.11%) was diagnosed with syphilis, with positive VDRL followed by positive fluorescent treponemal antibody absorption test (FTA-ABS). Our data showed a significantly higher prevalence of positive results for syphilis in our population (0.3%; 95%CI: 0.06–0.87%), but we did not register any cases of active disease because the 3 patients described were already diagnosed and treated.

Monich et al. 9 studied the seroprevalence of HIV, syphilis, HTLV, and hepatitis B and C among blood donors in Curitiba, PR, Brazil, between 2003 and 2012. Among 399,280 blood donations, the seroprevalence was 0.9%, 0.5%, 0.2%, 0.3%, and 0.8%, respectively.

When we evaluated the presence of hepatitis B immunization markers, we found a significant proportion of the patients (65.3%) not immune to the virus. Considering only vaccinated patients (with anti-HBs positive and anti-Hbc IgG negative results), the proportion of patients who were immunized was 31.5% (95%CI: 28.7–34.7%). Even though between 5 and 10% of adults who receive the 3-dose vaccination schedule do not present seroconversion, the rate of nonimmune patients is high, considering that the vaccine is part of the national adult immunization schedule, for an age range of 19 to 49 years old. 10

The correlation between absence of immunization and advancement of the age of the patients, as shown in Table 5 , also reflects the lack of a vaccine for hepatitis B before its invention (in 1982), in addition to the hepatitis B immunization policy of Brazil starting from 1989 and becoming part of the vaccine calendar in some states only in 1992.

The prevalence of HTLV among IVF patients in Sweden was described by Malm et al. 11 The recorded seroprevalence was 2.3 per 10,000 patients undergoing IVF in that country. Due to the smaller sample size in our study, it was impossible to compare the results with those of the Swedish study.

## Conclusion

All the seroprevalences were shown to be inferior to those reported in the compared studies. We found that a significant part of our IVF population was not immunized against hepatitis B. More attention should be paid to the guidance on vaccination for these patients. Despite the significant number of patients evaluated in the present study, more epidemiological studies are needed to better guide public practices and regulations to optimize results and available resources, because Brazil is a country that still experiences a shortage of financial resources.
